# Merkel Cell Polyomavirus Large T Antigen is Dispensable in G2 and M-Phase to Promote Proliferation of Merkel Cell Carcinoma Cells

**DOI:** 10.3390/v12101162

**Published:** 2020-10-14

**Authors:** Roland Houben, Marlies Ebert, Sonja Hesbacher, Thibault Kervarrec, David Schrama

**Affiliations:** 1 Department of Dermatology, Venereology und Allergology, University Hospital Würzburg, 97080 Würzburg, Germany; marliesebert@online.de (M.E.); Hesbacher_s@ukw.de (S.H.); Schrama_d@ukw.de (D.S.); 2 Department of Pathology, Centre Hospitalier Universitaire De Tours, INRA UMR 1282 BIP, 37200 Tours, France; thibaultkervarrec@yahoo.fr

**Keywords:** Merkel cell polyomavirus, large T antigen, cell cycle, Merkel cell carcinoma

## Abstract

Merkel cell carcinoma (MCC) is an aggressive skin cancer frequently caused by the Merkel cell polyomavirus (MCPyV), and proliferation of MCPyV-positive MCC tumor cells depends on the expression of a virus-encoded truncated Large T antigen (LT) oncoprotein. Here, we asked in which phases of the cell cycle LT activity is required for MCC cell proliferation. Hence, we generated fusion-proteins of MCPyV-LT and parts of geminin (GMMN) or chromatin licensing and DNA replication factor1 (CDT1). This allowed us to ectopically express an LT, which is degraded either in the G1 or G2 phase of the cell cycle, respectively, in MCC cells with inducible T antigen knockdown. We demonstrate that LT expressed only in G1 is capable of rescuing LT knockdown-induced growth suppression while LT expressed in S and G2/M phases fails to support proliferation of MCC cells. These results suggest that the crucial function of LT, which has been demonstrated to be inactivation of the cellular Retinoblastoma protein 1 (RB1) is only required to initiate S phase entry.

## 1. Introduction

Merkel cell carcinoma (MCC) is the most aggressive skin cancer [1]. It is considered to be a predominantly virus-induced disease because (i) the genome of the Merkel cell polyomavirus (MCPyV) can be detected in the vast majority of cases [2], (ii) a monoclonal integration of the virus in the genome of the tumor cells implies that integration occurred before tumor development, and (iii) MCPyV-positive MCC cells depend on expression of the virus-encoded T antigens [3].

Although sharing sequence homology with the well-studied simian virus (SV), 40 oncoproteins and the respective MCPyV T antigens exert rather different functions. In this respect, SV40 Large T antigen (LT) is a strongly transforming oncogene while SV40 small T (sT) has some supportive function in tumorigenesis [4]. In contrast, MCPyV-sT targets several pathways, which might be involved in MCC tumorigenesis [5], and demonstrates in vitro as well as in vivo transforming capacity [6,7] while, for MCPyV-LT transformation, could not be demonstrated so far, neither in standard in vitro transformation assays [6,8] nor in current mouse models [7,9]. Nevertheless, observations in human tumor cells still argue for an essential role of MCPyV-LT in MCC development. MCC tumor cell proliferation in vitro is dependent on LT function [10]. The MCC-associated LT proteins are always truncated due to stop codon mutations or incomplete integration [11,12], suggesting that some C-terminal domains need to be deleted to allow tumorigenesis. Notably, the fact that these deletions never affect the RB1-binding LxCxE motif [11,13] indicates that generally LT and specifically this motif are essential for MCC development.

The LxCxE motif is found in multiple cellular (e.g., HDAC1 and PRDM2) and viral proteins (e.g., adenovirus E1a, SV40-LT, and human papillomavirus E7) known to interact with members of the retinoblastoma protein family (RB1, p107, and p130) [14]. While SV40-LT can interact with all three family members [14], MCPyV-LT binds specifically to RB1, and inactivation of RB1 by MCPyV-LT is largely sufficient for its growth-supporting function in MCPyV-positive MCC cells [10].

In the current study, we wondered whether inactivation of RB1 by MCPyV-LT has to occur in MCC cells throughout the whole cell cycle or whether restricted presence of MCPyV-LT in certain cell cycle phases might be sufficient. Applying a system for cell-cycle-dependent degradation of LT, we demonstrate that expression of MCPyV-LT in G1 is as efficient as constitutive expression in rescuing LT-knockdown-induced growth suppression.

## 2. Materials and Methods

### 2.1. Cell Culture

The cell lines analyzed in this study include the MCPyV-positive MCC cell line MKL-1 [15] as well as HeLa cells derived from a cervix carcinoma [16]. HEK-293T cells [17] were used for lentivirus production. All cell lines were grown in RPMI 1640 supplemented with 10% FCS, 100 U/mL penicillin, and 0.1 mg/mL streptomycin.

### 2.2. Vectors

For inducible knockdown of MCPyV-LT, we used the lentiviral single vector TA.shRNA.tet, allowing constitutive green fluorescence protein (GFP) expression and Doxycycline (Dox)-inducible expression of an shRNA targeting all transcripts derived from the MCPyV early region [10]. The retroviral vector pIH [18] was used for expression of MCPyV-LT fusion proteins.

### 2.3. Retroviral Infection

Retroviral supernatants were produced in HEK293T cells using pHIT60 and pHIT456 as helper constructs. Two days following transfection, virus supernatants were filtered through 0.45-μm pore size filters, supplemented with 1 µg/mL polybrene, and added for overnight incubation to the cells. The next day, cells were washed with medium and then subjected to antibiotic selection.

### 2.4. Time Lapse Microscopy

Transduced HeLa cells were seeded in µ-Slides (Ibidi) and cultured in the presence of 5% CO_2_ at 37 °C. Phase contrast images and mRuby fluorescence were recorded in the course of time using a Nikon Eclipse Ti microscope.

### 2.5. Mixed Cell Culture Assay

Constitutive GFP expression from the TA.shRNA.tet construct was used to compare the growth behavior of double-infected and uninfected cells. TA.shRNA.tet cells were mixed with approximately 20% of untransduced cells, and changes in the frequency of GFP-positive TA.shRNA cells in the absence and presence of 1 µg/mL Doxycyclin (Dox) were recorded by flow cytometry over time.

*Immunoblotting*. Immunoblotting was performed as previously described [18]. The primary antibodies used in this study were directed against MCPyV-LT (CM2B4, Santa Cruz, CA, USA) or β-tubulin (TUB 2.1, Sigma-Aldrich, St. Louis, MO, USA).

### 2.6. Cell Cycle Analysis

Vybrant™ DyeCycle Violet (Thermo Fisher, Waltham, MA, USA) was used according to the manufacturer’s instructions to stain the DNA content of viable cells. Combined analyses of the DNA stain and mRuby was performed by flow cytometry.

## 3. Results and Discussion

Recently, we presented data demonstrating that MCPyV-LT-mediated inactivation of RB1 induces transcription of G2/M genes (e.g., *CCNB1* and *PLK1*) in MCC cells [10]. This observation raises the question whether RB1 inactivation in G2 is necessary for this gene induction or if it is a secondary effect mediated by proteins induced at the G1/S transition. In fact, regulation of G1/S genes mediated by RB family proteins affecting E2F transcription factors is by far the best investigated function of these tumor suppressors, and some authors suggested that, after the S-phase entry, the RB family proteins are relatively functionless until the next G1-phase [19]. This would imply that MCPyV-LT is only essential at the G1/S transition in MCC cells. However, others have shown that E2Fs exhibit functions beyond G1/S control [20], and that RB1 can play a role at the G2/M checkpoint [21,22,23]. In addition, a myriad of further RB1 interactors—in addition to E2Fs—have been described including many of which have no direct connection to the G1/S transition [24]. Such results raise the question whether the long-standing focus on the role of RB1/E2F in the control of the G1/S transition has given us a complete picture of the cellular changes that occur when RB1 is inactivated in cancer [24].

**Figure 1:**
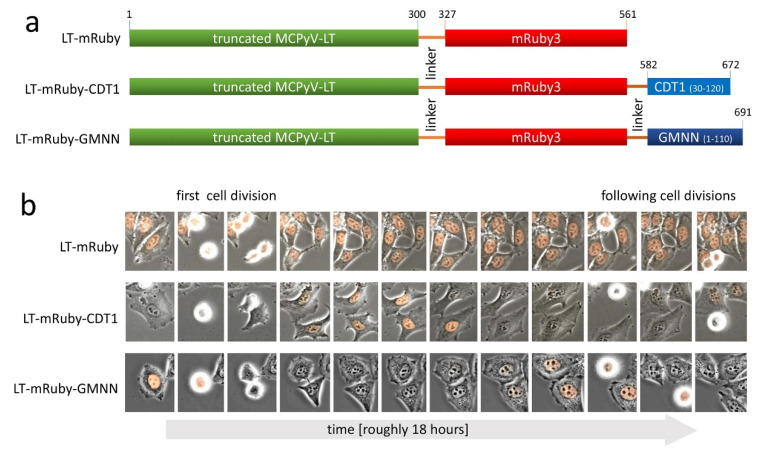
Merkel cell polyomavirus (MCPyV)- Large T antigen (LT) fusion proteins expressed in different phases of the cell cycle. (**a**) Schematic illustration of different Large T proteins analyzed in this study. To make SV40 Large T antigen (LT) detectable by flow cytometry and fluorescence microscopy, the red fluorescent protein mRuby3 was fused to the C-terminus of a truncated MCPyV-LT. To achieve cell cycle-dependent expression, either amino acids 30–120 of CDT1 or amino acids 1–110 of GMNN (also known as Geminin) were added at the C-terminus. (**b**) HeLa cells were stably transduced with retroviral vector pIH encoding the indicated LT variants depicted in (**a**). Time lapse fluorescence microscopy was performed, and the presence of mRuby fluorescence in the course of time is depicted.

To address one aspect of this question, we asked whether inactivation of RB1 by MCPyV-LT at the G1/S boundary in MCC cells might be sufficient to support growth of these cells. To this end, we designed MCPyV-LT fusion proteins, which are either expressed throughout the cell cycle, specifically in G1 or specifically in G2/M (Figure 1). To monitor such an expression pattern, we first cloned the coding sequence of the red fluorescent protein mRuby in frame with the sequence of the N-terminal 300 amino acids of MCPyV-LT. The two proteins were connected by a highly flexible glycine-serine (4xGGGGS) linker to preserve biological activity [25]. Furthermore, we adapted the fluorescent ubiquitination-based cell cycle indicator (FUCCI) system to achieve cell cycle-dependent expression of the LT-mRuby fusion protein. This system utilizes the tight proteasome-mediated degradation of the chromatin licensing and DNA replication factor 1 (CDT1), and its antagonist known as the DNA replication inhibitor GMNN (also known as Geminin) [26]. For degradation in G1 and G2 phases, GMNN and CDT1 fragments, respectively, were cloned in frame with LT-mRuby (Figure 1a). Time lapse fluorescence microscopy of HeLa cells transduced with these constructs revealed that the control LT-mRuby was constitutively expressed while LT-mRuby-CDT1 only started to be detectable shortly after cell division and disappeared again several hours before the next cell division (Figure 1b). In contrast, LT-mRuby-GMNN fluorescence was visible until cell division and disappeared directly thereafter, indicating degradation of the fusion protein in early G1 (Figure 1b).

**Figure 2:**
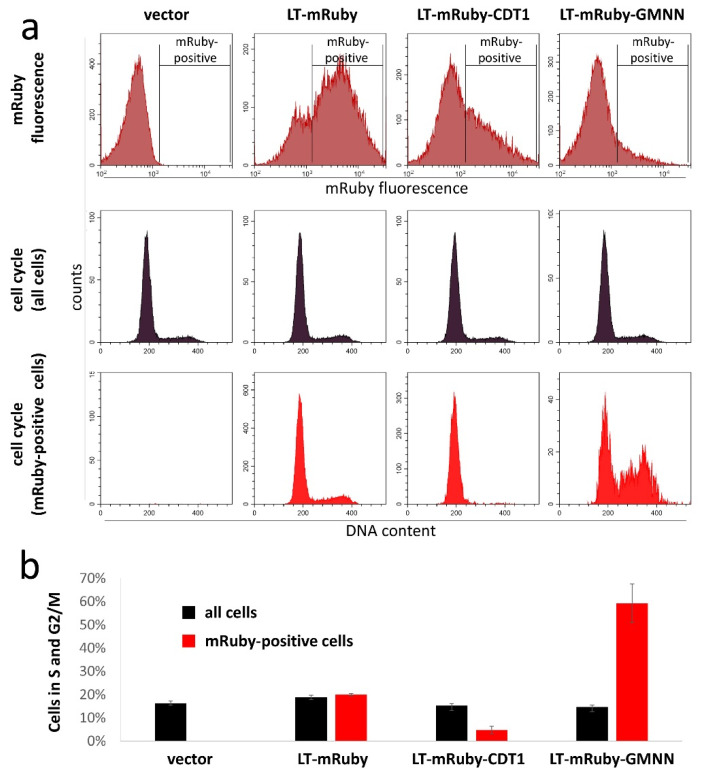
LT-mRuby-CDT1 is largely lacking in S and G2/M phases of the cell cycle in MKL-1 cells. MKL-1 TA.shRNA.tet cells were transduced with retroviral pIH vectors coding for the different indicated LT fusion proteins. Using Vybrant™ DyeCycle Violet, the DNA of viable cells was stained, and combined analyses of the DNA stain and mRuby fluorescence was performed by flow cytometry. (**a**) mRuby fluorescence is depicted in the upper, the cell cycle distribution of all cells in the middle, and of only mRuby-positive cells in the lower row. (**b**) The percentages of cells in S and G2/M phases of the total population and of only mRuby-positive cells are depicted as bar graphs (mean values (±SD) of at least three measurements).

To study the ability of LT to support growth of MCC cells in the different cell cycle phases, we next transduced the MCPyV-positive MKL-1 cells with these different LT-mRuby variants. To evaluate cell cycle specific expression in these spheroidal growing cells, we used Vybrant™ DyeCycle Violet, which is a dye allowing quantification of the DNA content of viable cells [27]. Combined analyses of the DNA stain and mRuby fluorescence by flow cytometry demonstrated that the mRuby-positive cells expressing LT-mRuby-CDT1 were mostly G1 cells while the LT-mRuby-GMNN expressing cells were highly enriched for S and G2/M cells (Figure 2). This suggests that, in MKL-1, cell cycle-specific degradation of LT fusion proteins largely worked as intended.

**Figure 3:**
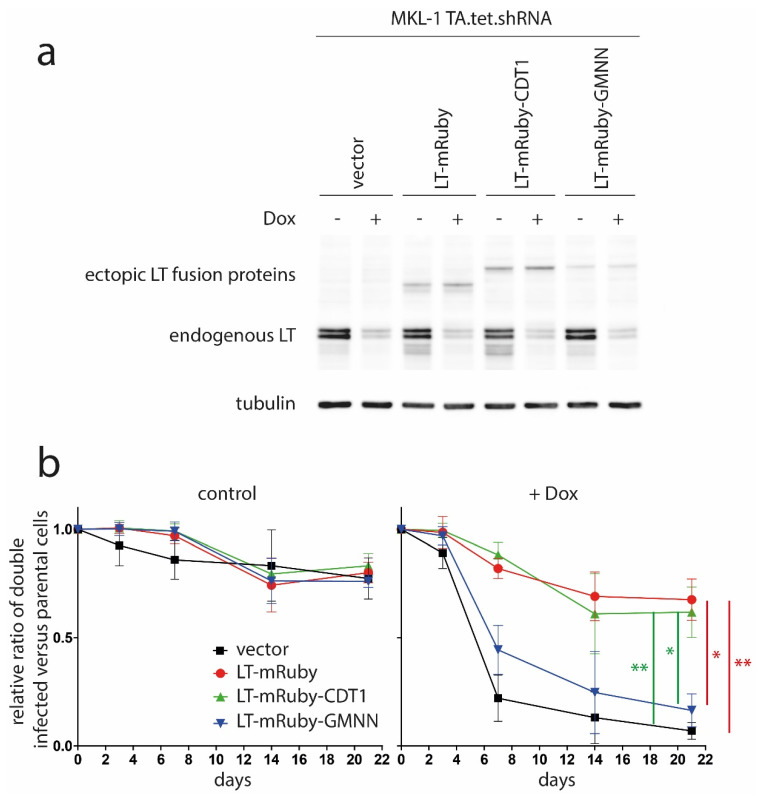
MCPyV-LT present only in G1 is capable of rescuing the growth arrest induced by knockdown of endogenous LT. MKL-1 cells transduced with a vector (TA.shRNA.tet) allowing Dox-inducible expression of a T antigen-targeting shRNA as well as constitutive expression of GFP were additionally transduced with retroviral vectors coding for the indicated LT fusion proteins. Notably, these ectopically-expressed LT variants had been rendered shRNA-insensitive by six silent nucleotide exchanges in the shRNA target sequence. (**a**) Following five days in the absence or presence of Dox (1 µg/mL) expression of endogenous and ectopic LT were analyzed by immunoblot. (**b**) The ratios of a mixed population of green-fluorescent double infected cells with uninfected, non-fluorescent parental cells were determined over time. Relative ratios based in each case on the measurement of the first time point were calculated and mean values (±SD) of three independent experiments are depicted. Statistical significance was evaluated by calculating the area under the curve (AUC) for each setting of the Dox-treated cells and then comparing the AUC of each group by one-way-ANOVA. Since the group means were significantly different (*p* = 0.2204), the Tukey post hoc test was performed to identify significant between-group differences. To this end, the comparison of LT-mRuby/vector, LT-mRuby/LT-mRuby-GMNN, LT-mRuby-CDT1/vector and LT-mRuby-CDT1/LT-mRuby-GMNN were statistically significant (* = adjusted *p* < 0.05; ** = adjusted *p* < 0.01).

Since the MKL-1 cells were also stably transduced with TA.shRNA.tet, which is a vector allowing doxycycline (Dox)-inducible expression of an shRNA targeting all MCPyV-T antigen mRNAs, the ectopic expression of the fusion proteins allowed us to study their biological activity in MCC cells. This was possible since the TA.shRNA binding site was destroyed by six silent mutations in the coding sequence of the ectopically expressed LT variants. Dox-induced knockdown of endogenous LT as well as expression of the ectopically expressed LT variants was monitored by an immunoblot (Figure 3a). Notably, the weaker band for LT-mRuby-GMNN—compared to LT-mRuby or LT-mRuby-CDT1—is expected due to a smaller percentage of the total cell population being in S and G2, which are the cell cycle phases where the protein is stably expressed (Figure 2a). In contrast, analysis of mRNA expression levels reveals that all three fusions constructs are transcribed at a similar level (Supplementary Figure S1; primers given in Table S1).

The impact on MCC cell proliferation of cell cycle phase-specific LT expression was then analyzed by flow cytometry in mixed cultures of green-fluorescent, double-infected cells and uninfected parental cells on the basis of GFP expression driven by the TA.shRNA.tet vector. To this end, Dox-induced knockdown of endogenous LT was associated with growth inhibition—evident by a gradual loss of GFP-positive cells over time—of TA.shRNA.tet cells additionally infected with an empty vector as well as cells expressing LT-mRuby-GMNN (Figure 3b). In contrast, in cells ectopically expressing LT-mRuby or LT-mRuby-CDT1, the growth arrest induced by knockdown of endogenous T antigens was largely reversed (Figure 3b). This was in accordance with previous experiments demonstrating rescue of TA.shRNA-induced growth arrest by only re-expressing LT. This indicates that, at least in this experimental system, LT function is sufficient even though the applied TA shRNA also targets sT [8,28]. More importantly, however, the fact that LT-mRuby-CDT1, whose expression is largely restricted to the G1 phase (Figure 1b and Figure 2), rescued as efficiently as constitutively expressed LT-mRuby strongly suggests that MCPyV-LT is dispensable for the G2/M transition in MCC cells. This was further confirmed by applying the EdU incorporation assay, demonstrating that LT-mRuby-CDT1 rescued reduced DNA synthesis as efficient as LT-mRuby (Supplementary Figure S2). Based on our previously published observation that inactivation of RB1 is fully sufficient to substitute for MCPyV-LT knockdown in MKL-1 cells [10], we can also conclude from the presented data that, for MCC cell proliferation, RB1 only has to be inactivated in G1 and not in later phases of the cell cycle. In this respect, real-time PCR analysis revealed that RB1 target gene expression is equally supported by LT-mRuby and LT-mRuby-CDT1 (Supplementary Figure S3).

It has to be noted that these observations do not exclude that inactivation of RB1 in cancer may have crucial consequences other than promoting the S-phase entry (e.g., regulation of apoptosis or differentiation status) [24], but with respect to driving the cell cycle, at least in the studied MCPyV-positive MCC cells, RB1 inactivation is only essential in G1. Notably, SV40-LT has been demonstrated to only bind unphosphorylated retinoblastoma protein present in GO/G1 [29,30]. Hence, it will be interesting to study whether binding of MCPyV-LT to RB1 is also restricted to the G0/G1 phase.

## Supplementary Materials

The following are available online at https://www.mdpi.com/1999-4915/12/10/1162/s1. Figure S1: The weaker band observed for LT-mRuby-GMNN (Figure 3) is not due to reduced transcription from the retroviral vector. Figure S2: LT-mRuby-CDT1 rescues reduced DNA synthesis upon knockdown of endogenous MCPyV-LT as efficient as LT-mRuby. Figure S3: LT-mRuby-CDT1 supports RB1 target gene expression as efficient as LT-mRuby. Table S1: Primers used for real time PCR.
