# Supplementary material for: Merkel Cell Polyomavirus Large T Antigen is Dispensable in G2 and M-Phase to Promote Proliferation of Merkel Cell Carcinoma Cells
Source: Viruses. 2020 Oct 14;12(10):1162. doi: 10.3390/v12101162 (PMC7602435; doi:10.3390/v12101162)
Supplement: Supplementary file 1 [file viruses-12-01162-s001.pdf]

viruses

MDPI

Supplementary Table S1: Primers used for Real Time PCR.

	forward	reverse
**LT-mRuby**	CGAAGCTCTGCAAGCTCTGC	TGACCCGCCTCCGCCTGA
**RPLP0**	CCATCAGCACCACAGCCTTC	GGCGACCTGGAAGTCCAACT
**MYB**	TCCACACTGCCCAAGTCTCT	AGCAAGCTGTTGTCTTCTTTGA
**CDC6**	CCTGTTCTCCTCGTGTAAAAGC	GTGTTGCATAGGTTGTCATCG

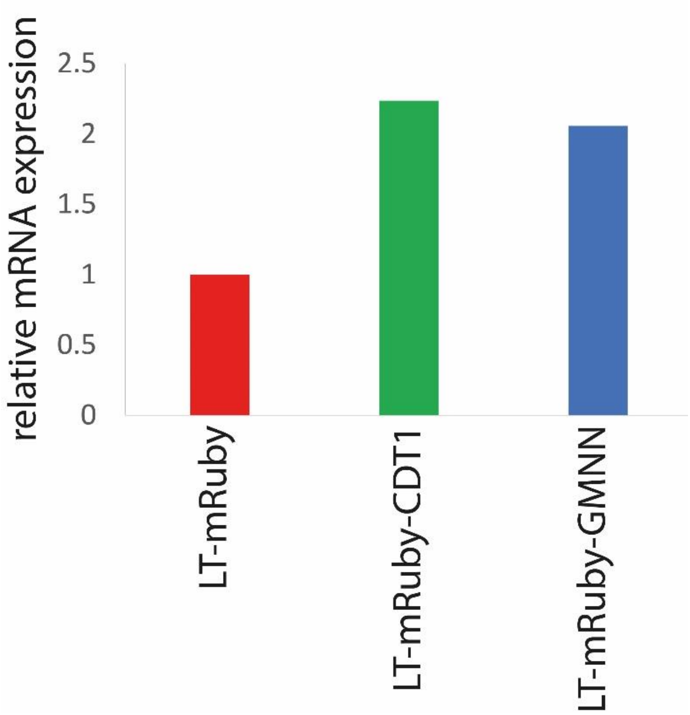

Figure S1. The weaker band observed for LT-mRuby-GMNN (Figure 3) is not due to reduced transcription from the retroviral vector. Total RNA was isolated from MKL1 cells transduced with the retroviral vector pIH encoding for the indicated LT fusion constructs. Following reverse transcription quantitative real time PCR was performed with SYBRGreen using primers specific for a sequence present in all three LT-fusion mRNAs. *RPLP0* served as internal reference. Relative expression was calculated using the e ΔΔCq method and LT-mRuby as calibrator.

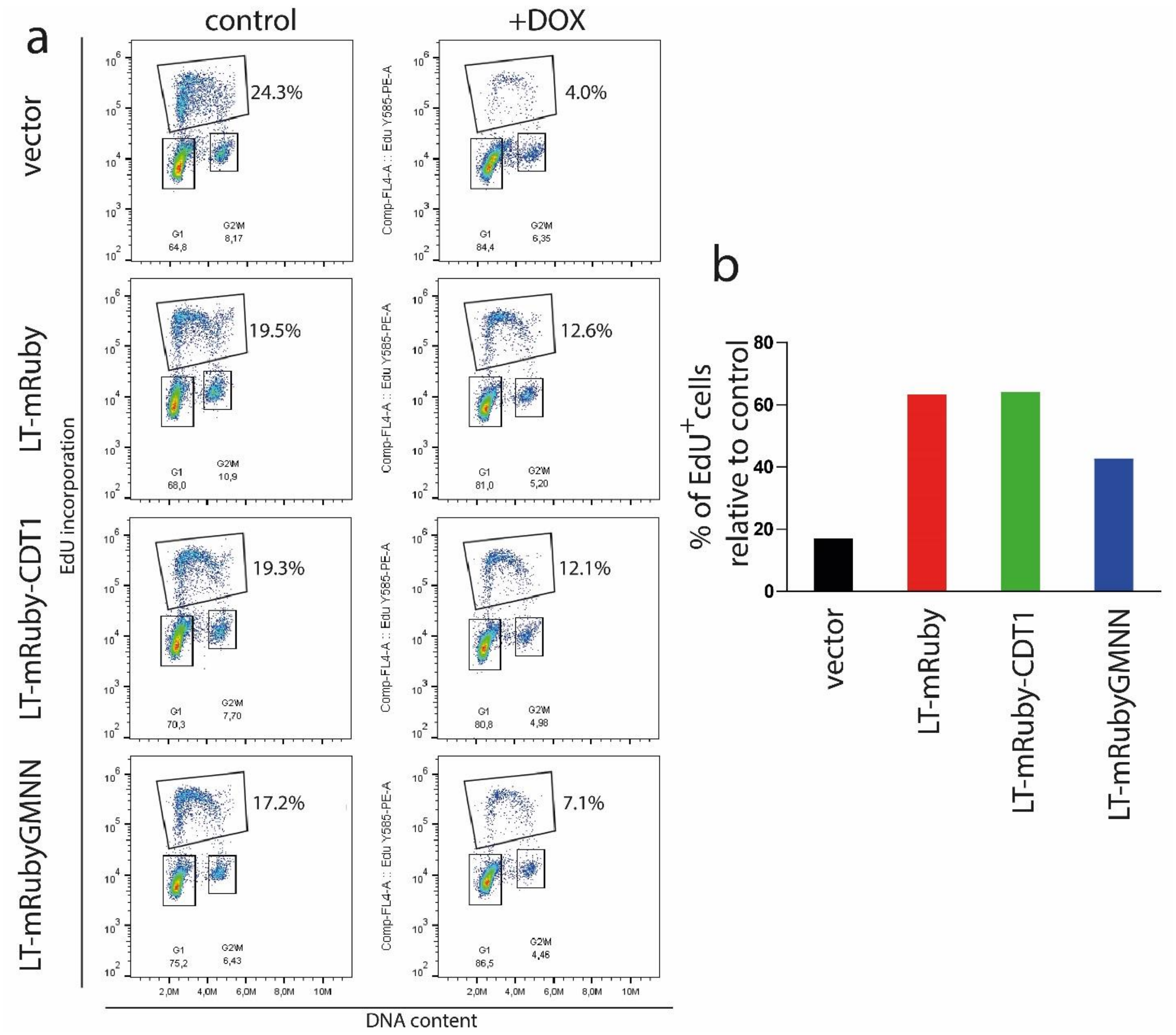

Figure S2. LT-mRuby-CDT1 rescues reduced DNA synthesis upon knockdown of endogenous MCPyV-LT as efficient as LT-mRuby. MKL-1 cells transduced with a vector (TA.shRNA.tet) allowing Dox-inducible expression of a T antigen-targeting shRNA were additionally transduced with retroviral vectors coding for the indicated LT fusion proteins. Notably, these ectopically-expressed LT variants had been rendered shRNA-insensitive by six silent nucleotide exchanges in the shRNA target sequence. Following five days in the absence or presence of Dox (1 µg/ml) the rate of DNA synthesis was analyzed using the EdU incorporation assay in combination with DNA staining with Hoechst 33342. a) Dot blots derived from flow cytometry analysis are given, and the percentage of EdU-positive cells is indicated. b) The percentage of Edu positive cells of the Dox-treated cells relative to the respective DMSO treated controls are given.

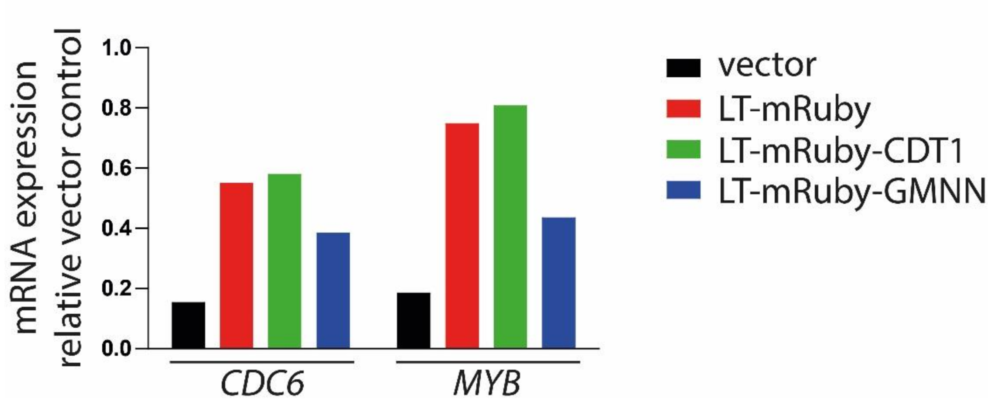

Figure S3. LT-mRuby-CDT1 supports RB1 target gene expression as efficient as LT-mRub**y.** MKL-1 cells transduced with a vector (TA.shRNA.tet) allowing Dox-inducible expression of a T antigen-targeting shRNA were additionally transduced with retroviral vectors coding for the indicated LT fusion proteins. Notably, these ectopically-expressed LT variants had been rendered shRNA-insensitive by six silent nucleotide exchanges in the shRNA target sequence. Following five days in the absence or presence of Dox total RNA was isolated and following reverse transcription, expression of the two RB1 target genes *CDC6* and *MYB* was analyzed by SYBRGreen real time PCR. RPLP0 served as internal reference. Expression is depicted as relative to the untreated vector control.
